# Conjunctival hyperaemia with the use of latanoprost versus other prostaglandin analogues in patients with ocular hypertension or glaucoma: a meta-analysis of randomised clinical trials

**DOI:** 10.1136/bjo.2007.135111

**Published:** 2008-11-19

**Authors:** F Honrubia, J García-Sánchez, V Polo, J M Martínez de la Casa, J Soto

**Affiliations:** 1 Ophthalmology Service, Hospital Miguel Servet, Zaragoza, Spain; 2 Ophthalmology Service, Hospital Clínico, Madrid, Spain; 3 Medical Unit, Pfizer Spain, Madrid, Spain

## Abstract

**Aim:**

To conduct a meta-analysis of randomised clinical trials (RCTs) in order to evaluate the development of conjunctival hyperaemia after the use of latanoprost versus travoprost and bimatoprost, in patients with ocular hypertension or glaucoma.

**Methods:**

In order to identify the potentially relevant RCTs, a systematic literature retrieval was conducted in Medline, Embase and Cochrane Controlled Trials Register (1995–April 2007) databases The outcome measure was the appearance of conjunctival hyperaemia during the study. Statistical analyses included the calculation of odds ratio (OR) and its respective confidence interval, along with intertrial statistical heterogeneity. Publication bias was evaluated through a funnel plot, and a sensitivity analysis was also performed.

**Results:**

In total, 13 RCTs involving 2222 patients with ocular hypertension or glaucoma were included, five comparing latanoprost versus travoprost, seven comparing latanoprost versus bimatoprost and one comparing latanoprost versus travoprost and bimatoprost. The combined results showed that latanoprost produced lower occurrence of conjunctival hyperaemia than both travoprost (OR = 0.51; 95% CI 0.39 to 0.67, p<0.0001) and bimatoprost (OR = 0.32; 95% CI 0.24 to 0.42, p<0.0001). No significant heterogeneity was found between the included RCTs. There was no evidence of publication bias. In the sensitivity analysis performed, none of the clinical trials included in this meta-analysis has an important impact in the global estimation of OR.

**Conclusions:**

According to available data, the use of latanoprost is associated with a lower incidence of conjunctival hyperaemia when compared with travoprost and bimatoprost in the treatment of patients with ocular hypertension or glaucoma.

The estimated prevalence of glaucoma is 2% in the general population, 3% in people over 50, 5.7% in subjects 73 and 74 years old and 23.2% in those aged 75 years and older. This number is expected to increase in the future, given the progressive ageing of the population.^1^^–^3

For patients with ocular hypertension or open-angle glaucoma, drug therapy focuses on reducing intraocular pressure (IOP) levels in order to delay or prevent the progression of ocular hypertension to glaucoma, and to slow disease progression in glaucoma patients.^4^ ^5^ In both cases, patients require lifelong treatment and follow-up care to preserve vision, so it is essential long-term patient compliance and medication persistency, because those who do not continue therapy risk developing elevated IOP levels and, over time, progressing to blindness.^6^

Compliance and persistency depends on many factors, including patient satisfaction with medication, medication costs, ease of medication administration and patient understanding of the importance of taking their medication over the long term, although one of the most important factors is local and systemic side effects.^7^

Currently, first-line treatment usually consists of monotherapy with a topical hypotensive drug. Although ophthalmologists traditionally have prescribed beta-blockers as first-line ocular hypotensive therapy, due to the possibility of producing systemic side effects, other therapeutic options are currently preferred, with prostaglandin analogues being one of the most widely used.^8^

Topical prostaglandins such as latanoprost, bimatoprost and travoprost are similar in that they require once-daily instillation, produce few systemic side effects and reduce IOP levels similarly.^9^^–^11 However, some prostaglandin-treated patients experience conjunctival hyperaemia,^12^ and this condition is of concern because this side effect may have a negative affect on whether the patient takes the drug as directed (compliance) and/or continues to use the drug over time (persistency).

Although a lower rate of conjunctival hyperaemia has been reported with latanoprost in contrast to bimatoprost and travoprost,^13^ no systematic review and meta-analysis has examined this issue. Therefore, the aim of this work was to conduct a meta-analysis of RCTs comparing latanoprost against bimatoprost and travoprost, either together or in separated studies, in patients with ocular hypertension and/or glaucoma.

## METHODS

### Search strategy

Reports of RCTs comparing latanoprost, bimatoprost and travoprost were identified through a systematic search. A computerised literature search was conducted in Medline, Embase and Cochrane Controlled Trials Register databases from 1995 to April 2007 for relevant articles in English.

We used the Medical Subject Heading and the following key words: glaucoma, ocular hypertension, randomisation, trial, latanoprost, bimatoprost, travoprost and conjunctival/ocular hyperaemia. References from the reviewed articles were also searched for relevant titles.

### Study selection

Two reviewers independently conducted the literature search and extraction of relevant articles. The title and abstract of potentially relevant studies and review articles were screened for appropriateness before retrieval of the full articles.

The following selection criteria were used to identify published studies for inclusion in this meta-analysis: (a) study design—RCTs in adults (age>18 years); (b) population—patients with ocular hypertension and/or glaucoma; (c) intervention—latanoprost versus other prostaglandins analogues (bimatoprost or travoprost) as monotherapy; (d) outcome variable—conjunctival hyperaemia. These articles were written in English. Abstracts from conferences without raw data available for retrieval and duplicate publications were excluded.

### Data extraction

Two reviewers performed separately the data extraction and methodological quality assessment of trials that were included. The reviewers were blinded for the names of the authors and their institution, the names of the journals, sources of funding and acknowledgments. Any disagreements between the reviewers were resolved by discussion to reach consensus. A third reviewer was involved when required.

A customised form was created to record the information of selected articles: year of publication, information of study design (double-blind, parallel or crossover), length of study, number of subjects, age, sex, type of glaucoma and proportion of conjunctival hyperaemia.

The primary outcomes measure was the incidence of conjunctival hyperaemia over treatment visits. The reason for exclusion was recorded on a standard form. Excluded publications were reassessed to ensure that all eligible publications were included.

### Assessment of study quality

Two reviewers independently rated study quality using the Jadad instrument for the assessment of the quality of trials reports.^14^ This instrument is a point scale ranging from 0 to 8, with points derived from the description of randomisation, blinding, inclusion and exclusion criteria, withdrawals and method of assessing adverse events.

### Statistical methods and assessment of heterogeneity

The statistical analysis was carried out by Comprehensive Meta-Analysis software version 2.2 (Biostat, Englewood Cliffs, New Jersey) (http://www.meta-analysis.com).

For dichotomous outcomes, we calculated a pooled odds ratio (OR) and 95% CIs. The OR was defined as the odds of an outcome in those who received latanoprost therapy compared with the odds in those who received bimatoprost or travoprost. The ORs of different RCTs were combined by using the fixed effects model of Mantel and Haenszel^15^ and the random effects model of Der Simonian and Laird.^16^

Intertrial statistical heterogeneity was explored using the Cochran Q test with calculated I^2^,indicating the percentage of the total variability in effect estimates among trials that is due to heterogeneity rather than to chance.^17^ I^2^ values of 50% or more indicate a substantial level of heterogeneity. Publication bias was assessed by visually inspecting a funnel plot.

All p values were two-sided with statistical significance set at an α level of 0.05. We followed the Quality of Reporting Meta-analysis guidelines for reporting and discussing these meta-analytical results.^18^

To exclude the possibility that any one study was exerting excessive influence on the results, we conducted a sensitivity analysis by systematically excluding each study at a time and then rerunning the analysis to assess the change in ORs.

## RESULTS

### Literature search

There were 31 articles relevant to the search term. A total of 18 potential RCTs of latanoprost versus other prostaglandin analogues were identified through the literature search,^19^^–^36 five comparing latanoprost vs travoprost, seven comparing latanoprost vs bimatoprost and one comparing latanoprost vs bimatoprost and travoprost.

**Figure 1:**
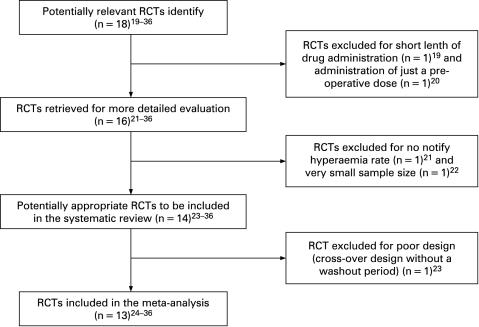
Selection algorithm for the randomised controlled trials (RCTs) included in this meta-analysis.

Finally, 13 articles involving 2222 patients with ocular hypertension or glaucoma were included in this meta-analysis.^24^^–^36 The algorithm flow chart for the selection of RCTs to be included in our analysis is shown in fig 1.

### Characteristics of trials

Table 1 shows the characteristics of the RTCs that were included in this meta-analysis. Overall, 2222 patients were evaluated for an average period of follow-up of 4.1 months (min = 2 weeks; max = 9 months). Nine hundred and thirty-one patients were treated with latanoprost, 624 with bimatoprost and 667 with travoprost.

**Table 1:** Characteristics of 13 trials meeting criteria for inclusion in the meta-analysis

Source	Design	Intervention	Duration of study	No of patients	Percentage of patients with hyperaemia			Jadad Score
					LAT	BIMAT	TRAVO	
Gandolfi *et al*^24^	Parallel	LAT vs BIMAT	3 months	LAT = 113; BIMAT = 119	14.2	36.1		7
Dubiner *et al*^25^	Parallel	LAT vs BIMAT	1 month	LAT = 22; BIMAT = 21	13.6	14.3		5
Noecker *et al*^26^	Parallel	LAT vs BIMAT	6 months	LAT = 136; BIMAT = 133	20.6	44.4		7
Walters *et al*^27^	Parallel	LAT vs BIMAT	1 month	LAT = 38; BIMAT = 38	15.8	39.5		6
Konstas *et al*^28^	Crossover	LAT vs BIMAT	7 weeks each treatment	LAT = 21; BIMAT = 21	28.6	71.4		5
Dirks *et al*^29^	Parallel	LAT vs BIMAT	3 months	LAT = 27; BIMAT = 33	7.4	21.2		6
Konstas *et al*^30^	Crossover	LAT vs BIMAT	3 months each treatment	LAT = 123; BIMAT = 123	7.3	26		7
Netland *et al*^31^	Parallel	LAT vs TRAVO	12 months	LAT = 193; TRAVO = 402	27.6		44	6
Parrish *et al*^32^	Parallel	LAT vs TRAVO vs BIMAT	3 months	LAT = 136; BIMAT = 136; TRAVO = 138	47.1	68.6	58	7
Parmaksiz *et al*^33^	Parallel	LAT vs TRAVO	9 months	LAT = 16; TRAVO = 18	6.2		38.8	5
Chen *et al*^34^	Parallel	LAT vs TRAVO	3 months	LAT = 36; TRAVO = 37	8.3		13.5	6
Garcia-Feijoo *et al*^35^	Parallel	LAT vs TRAVO	2 weeks	LAT = 30; TRAVO = 32	3.3		6.2	5
Konstas *et al*^36^	Crossover	LAT vs TRAVO	8 weeks each treatment	LAT = 40; TRAVO = 40	15		37.5	6

BIMAT, bimatoprost; LAT, latanoprost; TRAVO, travoprost.

Patients’ ages ranged from 58 to 73 years with a mean of 65; 41.4% were men, 1364 patients (61.4%) suffered from open-angle glaucoma, 678 (30. 5%) from ocular hypertension and 180 (8.1%) from another type of glaucoma (chronic angle-closure glaucoma, exfoliative glaucoma and pigmentary glaucoma).

The proportion of patients treated with latanoprost who developed conjunctival hyperaemia was 16.5% (min = 3.3%; max = 47.1%), in the bimatoprost group 40.2% (min = 14.3%; max = 68.6%) and in the travoprost group 33% (min = 6.2%; max = 58%).

### Hyperaemia outcome

**Figure 2:**
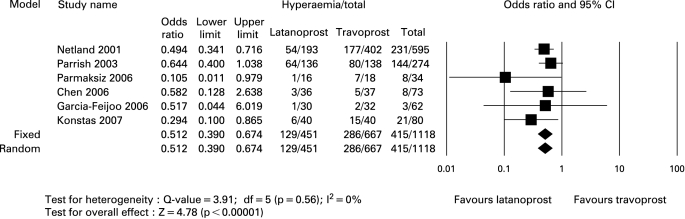
Global and partial statistical data of clinical trials comparing latanoprost and travoprost.

The combined results of different clinical trials suggested that the use of latanoprost 0.005% caused a lower percentage of appearance of conjunctival hyperaemia compared with travoprost 0.004% (OR = 0.51; 95% CI 0.39 to 0.67, p<0.00001). No significant heterogeneity was found between included clinical trials (Q = 3.98; p = 0.56) (fig 2).

**Figure 3:**
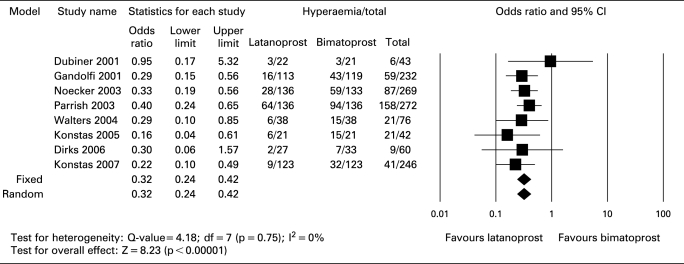
Global and partial statistical data of clinical trials comparing latanoprost and bimatoprost.

Moreover, the results of this meta-analysis also showed that the utilisation of latanoprost 0.005% is associated with a lower development of conjunctival hyperaemia when compared with bimatoprost 0.003% (OR = 0.32; 95% CI 0.24 to 0.42, p<0.00001). Heterogeneity between included clinical trials did not show any significance (Q = 4.18; p = 0.75) (fig 3).

### Sensitivity analysis

In order to analyse the consistency and robustness of the results, a sensitivity analysis was performed (table 2). First, the results obtained were compared using the fixed and random models. Changing the model from fixed to random effects did not change the results of our meta-analysis.

**Table 2:** Sensitivity analysis of the association of conjunctival hyperaemia and the use of latanoprost, travoprost and bimatoprost

Latanoprost vs bimatoprost			
Statistical model	No of studies	No of patients	OR (95% CI)
Fixed effects	8	1240	0.32 (0.24 to 0.42)
Random effects	8	1240	0.32 (0.24 to 0.42)
Analyses excluding			
Dubiner *et al*^25^	7	1197	0.31 (0.24 to 0.41)
Gandolfi *et al*^24^	7	1008	0.33 (0.24 to 0.44)
Noecker *et al*^26^	7	971	0.32 (0.23 to 0.44)
Parrish *et al*^32^	7	968	0.29 (0.21 to 0.40)
Walters *et al*^27^	7	1164	0.32 (0.24 to 0.43)
Konstas *et al*^28^	7	1198	0.33 (0.25 to 0.43)
Dirks *et al*^29^	7	1180	0.32 (0.24 to 0.42)
Konstas *et al*^30^	7	994	0.34 (0.25 to 0.45)

Latanoprost vs travoprost			
Statistical model	No of studies	No of patients	OR (95% CI)
Fixed effects	6	1118	0.51 (0.39 to 0.67)
Random effects	6	1118	0.51 (0.39 to 0.67)
Analyses excluding			
Netland *et al*^31^	5	523	0.54 (0.36 to 0.80)
Parrish *et al*^32^	5	844	0.46 (0.33 to 0.64)
Parmaksiz *et al*^33^	5	1084	0.53 (0.40 to 0.69)
Chen *et al*^34^	5	1045	0.51 (0.39 to 0.67)
Garcia-Feijoo *et al*^35^	5	1056	0.51 (0.39 to 0.68)
Konstas *et al*^36^	5	1038	0.53 (0.40 to 0.71)

Second, in order to assess the influence of each individual clinical trial included in the meta-analysis, each study was excluded at a time and the analysis performed again to determine the change in the OR. The punctual estimators for OR vary between 0.46 and 0.54 in the latanoprost–travoprost analysis, and between 0.29 and 0.34 in the latanoprost–bimatoprost analysis after excluding one by one each original clinical trial. None of the clinical trials included in this meta-analysis had an important impact in the global estimation of the OR.

### Publication bias

**Figure 4:**
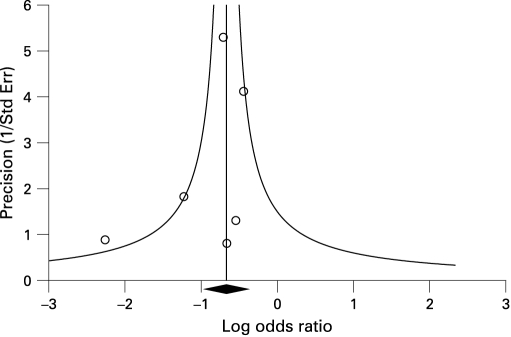
Funnel plot of clinical trials included in the meta-analysis comparing latanoprost vs travoprost.

**Figure 5:**
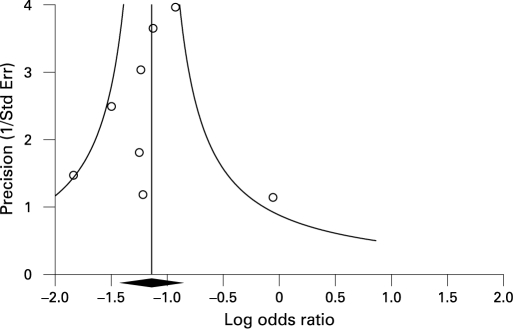
Funnel plot of clinical trials included in the meta-analysis comparing latanoprots vs bimatoprost.

An analysis of publication bias was conducted. For each separate analysis (latanoprost–travoprost and latanoprost–bimatoprost), based on a visual analysis of the funnel plots, no evidence of publication bias was found (figs 4, 5).

## DISCUSSION

The hypotensive prostaglandin analogues are a novel class of intraocular-lowering medications used primarily for the treatment of glaucoma. In recent years, prostaglandins have emerged as the mainstay of treatment for ocular hypertension and/or glaucoma. These include latanoprost, travoprost and bimatoprost, which are ester or amide pro-drug analogues of the prostaglandin F2-alpha.

The efficacy in lowering IOP of the three compounds is very similar, and in a recent meta-analysis it was found that the difference in decreasing IOP between the three products was very small.^37^ However, according to the literature, it has been described that conjunctival hyperaemia occurs more frequently with either bimatoprost and travoprost than with latanoprost,^12^ ^13^ ^38^ although neither systematic review nor meta-analysis has been performed to date to assess this issue properly. The reason for the reduction in hyperaemia caused by latanoprost in the eye compared with bimatoprost and travoprost likely lies in the latanoprost molecule and its pharmacological receptor profile.^39^

This meta-analysis was aimed at comparing the development of conjunctival hyperaemia of three prostaglandin analogues on the information reported in the international literature, as conjunctival hyperaemia is a condition of concern, since local side effects may have a negative affect on whether the patient takes the drug as directed (compliance) and/or continues to use the drug over time (persistency). Other local side effects such as the change in iris colour and the development of darker and longer eye lashes may also cause a decrease in treatment compliance and persistency, so it will be necessary to explore this issue in the future by conducting new studies.

The results of this meta-analysis show that the use of latanoprost is associated with a lower incidence of conjunctival hyperaemia. Arcieri *et al*^40^ found that there was a significant increase in hyperaemia scores in the latanoprost, bimatoprost and travoprost groups 1 week after baseline. Hyperaemia scores reached their peak 15 days after baseline and started to decrease 1 month after therapy was initiated. Thus, it is important to point out that the conjunctival hyperaemia could decrease during the use of prostaglandin analogues in daily medical practice.

This meta-analysis may have some limitations. First, we cannot fully exclude publication bias, because there were no sufficient studies to detect asymmetry in a funnel plot, and we did not perform a statistical test for the detection of publication bias: these tests have a very low power in meta-analysis of a small number of trials. In addition, we did not attempt to gain access to unpublished results, and only publications written in English were accepted. Second, clinical trials included in this meta-analysis were undertaken in many different countries, so we cannot eliminate location bias. Third, the studies included were heterogeneous in terms of study population, length of each study, number of patients of different studies, basal condition, associated comorbidities and the way of evaluating conjunctival hyperaemia. Access to individual level data could certainly have improved the quality of adjustment as well as the precision of estimates.

## CONCLUSION

The results of this meta-analysis suggest that latanoprost is associated with a lower incidence of conjunctival hyperaemia versus the use of bimatoprost and travoprost in the treatment of ocular hypertension and/or glaucoma. This information may be useful for determining the optimal treatment strategy for individual patients.

More research is needed to determine the incidence of conjunctival hyperaemia after the use of prostaglandin analogues in the mid and long term, as well as in real-world daily medical practice.
